# Depression mediated sleep disturbances and cognitive decline in Parkinson’s disease patient

**DOI:** 10.3389/fnins.2026.1722979

**Published:** 2026-01-29

**Authors:** Huashuo Zhao, Yi Sun, Qiushuang Wang, Sizhao Li, Zixuan Zhao

**Affiliations:** 1 School of Public Health, Xuzhou Medical University, Xuzhou, Jiangsu, China; 2 School of Public Health, Zhengzhou University, Zhengzhou, Henan, China; 3 Fengxian People’s Hospital, Shanghai, China; 4 School of Health Economics and Management, Nanjing University of Chinese Medicine, Nanjing, Jiangsu, China

**Keywords:** cognitions, depression, mediation, Parkinson’s disease, sleep disorders

## Abstract

**Objective:**

This study investigated the distinct mechanistic pathways linking depression, sleep disturbances, and cognitive decline in Parkinson’s disease, with emphasis on differential roles of daytime sleepiness versus rapid eye movement sleep disorder.

**Participants:**

The study included 450 Parkinson’s disease patients enrolled in the Parkinson’s Progression Markers Initiative (PPMI) between 2010 and 2024, leveraging longitudinal clinical and biomarker data.

**Measurements:**

Depressive symptoms (Geriatric Depression Scale, GDS), sleep disturbances (Epworth Sleepiness Scale, ESS for daytime sleepiness; REM Sleep Behavior Disorder Questionnaire, RBDSQ), and cognition (Montreal Cognitive Assessment, MoCA) were analyzed using mixed-effects models and structural equation modeling (SEM) with bootstrapped mediation.

**Results:**

Structural equation modeling demonstrated excellent model fit (CFI/TLI > 0.94; RMSEA < 0.08). Analyses revealed distinct pathological pathways: (1) Excessive daytime sleepiness impaired cognition fully through depression mediation (indirect effect *β* = −0.084, 95%CI [−0.097,-0.028], 66.1% mediated), while (2) REM sleep behavior disorder showed both direct negative effects on cognition (*β* = −0.116, *p* = 0.035) and depression-mediated effects (indirect effect β = −0.071, 95%CI [−0.110,-0.029], 38.2% mediated). These results highlight different intervention targets - treating depressive symptoms may fully mitigate cognitive impacts of daytime hypersomnia, while RBD may require both antidepressant and direct neuroprotective approaches.

**Conclusion:**

Divergent neurobehavioral mechanisms underlie sleep-related cognitive decline in PD: daytime sleepiness operates through mood dysregulation, while rapid eye movement sleep disorder involves additional direct neural insult. Interventions targeting these pathways may require distinct strategies.

## 1 Introduction

In patients with Parkinson’s disease (PD), cognitive dysfunction, as an important non-motor symptom, significantly affects the quality of life and disease prognosis (Zhu et al., 2021; Schneider and Kortagere, 2022; Kenangil et al., 2023). Studies have shown that a large number of PD patients eventually develop dementia, and their cognitive impairment is closely related to sleep disorders and depressive symptoms (Wang et al., 2022; Schenck et al., 2013; Postuma et al., 2010; Kang et al., 2016).

The core neuropathological features of PD are the progressive loss of dopaminergic neurons in the midbrain and the abnormal aggregation of *α*-synuclein (Tolosa et al., 2021). However, the clinical manifestations of PD extend well beyond its hallmark motor symptoms. Accumulating evidence indicates that non-motor symptoms often emerge early in the disease course and profoundly impact patients’ prognosis and quality of life. Among these, rapid eye movement (REM) sleep disorder (RBD), depressive symptoms, and cognitive impairment constitute a highly comorbid and interrelated triad of non-motor features. Studies have shown that a substantial proportion of individuals with PD eventually develop Parkinson’s disease dementia (PDD) (Gibson et al., 2024), and both RBD and depression not only serve as early warning signs of cognitive decline but may also accelerate disease progression through shared or interacting neurobiological mechanisms.

In the context of PD, the triad of symptoms comprising sleep disturbances, depression, and cognitive decline has a distinct pathophysiological basis and clinical significance. Unlike in the general aging population, these symptoms in PD likely stem from the widespread propagation of *α*-synuclein pathology, which affects not only the nigrostriatal system, but also key brain regions involved in sleep regulation (e.g., the locus coeruleus and pontine tegmentum), the limbic system, and cortical areas (Yuan et al., 2025). For instance, RBD frequently precedes the onset of motor symptoms by several years, often by more than a decade, and is now widely recognized as a prodromal marker of synucleinopathies (Feuerstein and Amara, 2023). Similarly, the frequent co-occurrence of depression and cognitive impairment may reflect early involvement of limbic–cortical circuits (Weintraub and Irwin, 2022). Moreover, the rate of conversion from mild cognitive impairment to dementia in PD is markedly faster than in other neurodegenerative conditions such as Alzheimer’s disease, suggesting that the dynamic interplay among sleep, mood, and cognition exhibits unique, disease-accelerating properties within the PD pathological framework.

Although existing studies have independently demonstrated that either sleep disturbances or depression can predict cognitive decline, there remains a paucity of research systematically examining their interactive effects, particularly through mediation analyses, in PD populations. Notably, empirical investigations grounded in PD-specific pathophysiology are lacking, especially those exploring how distinct sleep disorders, such as excessive daytime sleepiness (EDS) and RBD, may indirectly influence cognitive function via depressive symptoms. Importantly, models of sleep–mood–cognition relationships derived from general elderly cohorts may not be directly applicable to PD patients, given fundamental differences in underlying neurodegenerative processes, patterns of neurotransmitter dysfunction, and trajectories of symptom evolution. Therefore, large-scale longitudinal studies in well-characterized PD cohorts are urgently needed to conduct etiologically informed pathway analyses. Such efforts are essential for clarifying the mediating role of depression in the association between specific sleep disturbances and cognitive decline, thereby providing a robust theoretical foundation for the early identification of high-risk individuals and the development of precision interventions.

Accordingly, this study aims to systematically evaluate the independent and combined effects of EDS and RBD on cognitive function in a well-characterized cohort of patients with PD, and to examine the potential mediating role of depressive symptoms in these associations using mediation analysis. By elucidating the mechanisms underlying the “sleep–mood–cognition” pathway within the context of PD-specific pathophysiology, we hope to provide empirical evidence to support early identification of individuals at high risk for cognitive decline and inform the development of multi-targeted intervention strategies.

## 2 Materials and methods

### 2.1 Data sources

We obtained the research data from the publicly available Parkinson’s Progression Markers Initiative (PPMI) database (data download date: November 25, 2024; database URL;^1^ RRID: SCR_006431). PPMI is a large-scale, multicenter, prospective, international observational cohort study initiated by the Michael J. Fox Foundation with the primary aim of identifying reliable biomarkers associated with disease progression in individuals newly diagnosed with Parkinson’s disease who have not yet received dopaminergic therapy. Launched in 2010, the study has enrolled over 4,000 participants across nearly 50 clinical sites worldwide, including PD patients, at-risk individuals (e.g., carriers of LRRK2 or GBA mutations and those with REM sleep behavior disorder), and healthy controls, all of whom undergo long-term follow-up. The core PD cohort receives standardized assessments at baseline, 6 months, 12 months, and annually thereafter (e.g., at months 24, 36, 48, 60, etc.), encompassing clinical evaluations, neuroimaging, biospecimen collection, and cognitive testing.

**Figure 1:**
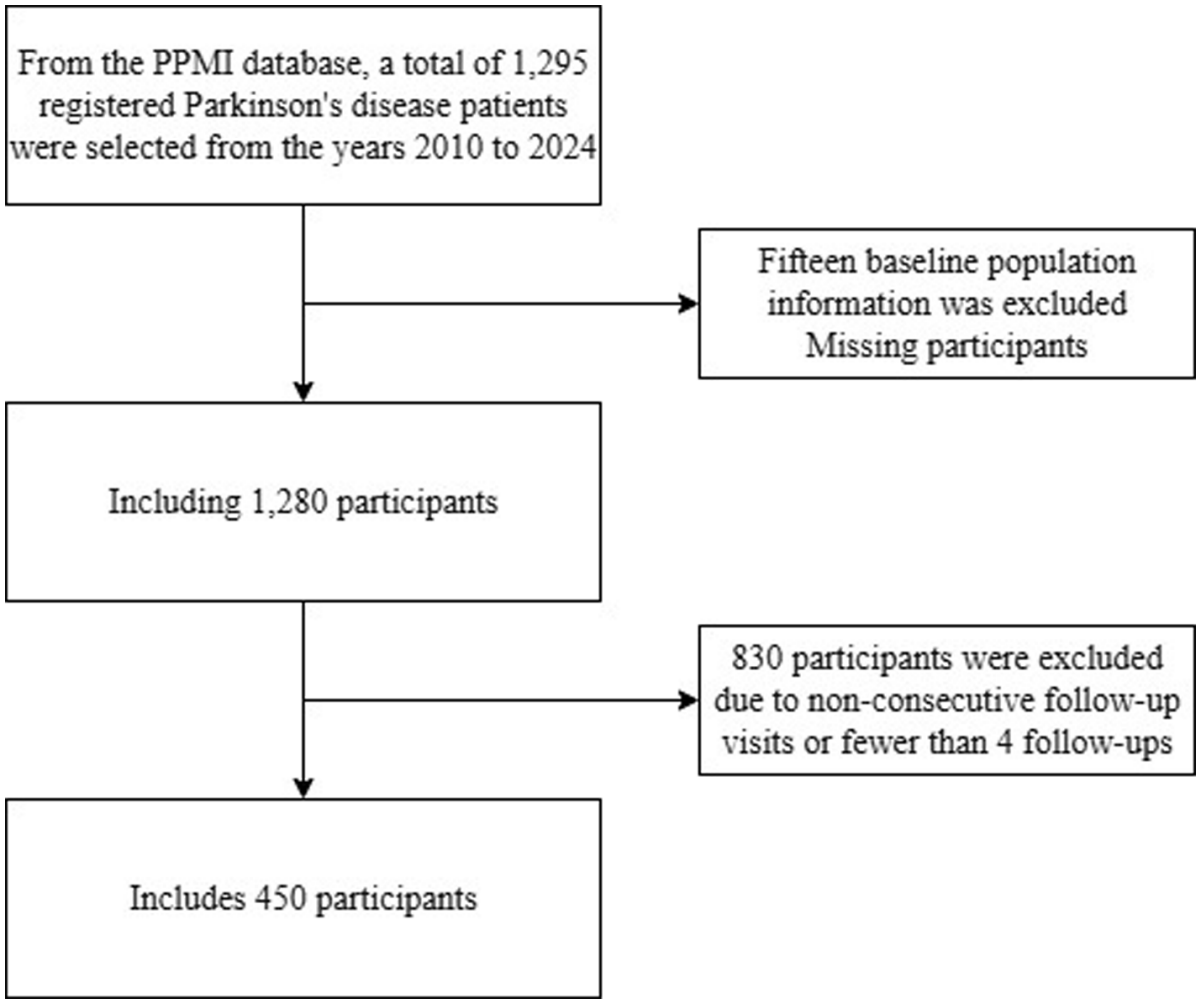
Flow chart of participant inclusion.

For this study, we included 450 PD patients registered in the PPMI database between 2010 and 2024 who met the predefined inclusion criteria. All participants completed longitudinal follow-up from baseline through at least month 60 (i.e., 5 years), with key assessment timepoints at baseline, 12, 24, 36, 48, and 60 months (see Figure 1 for details).

### 2.2 Clinical evaluation and scales

The Geriatric Depression Scale (GDS), developed by Yesavage et al. in 1982, is a specialized tool designed to assess the severity of depressive symptoms in older adults, addressing limitations in other depression measurement tools when applied to elderly populations (Shin et al., 2019). In this study, participants with a GDS score ≥ 5 were considered to exhibit signs of depression. The Montreal Cognitive Assessment Scale (MoCA) serves as a rapid screening tool for mild cognitive impairment, covering multiple cognitive domains including attention, executive function, memory, language, visuospatial abilities, abstract thinking, calculation, and orientation, with a total score of 30 points (Nasreddine et al., 2005). The Parkinson’s Progression Markers Initiative applied the following rule to adjust the raw Montreal Cognitive Assessment scores for years of education: if a participant’s education duration (EDUCYRS) was ≤12 years and their unadjusted MoCA score was <30, one point was added to the raw score to yield an education-adjusted MoCA score. No adjustment was made if the participant had more than 12 years of education or if the unadjusted MoCA score was already 30. The Rapid Eye Movement Sleep Behavior Disorder Screening Questionnaire (RBDSQ) is a self-report questionnaire with 10 items assessing RBD-related symptoms such as dream enactment behaviors and nighttime injuries. Scoring ranges from 0 to 13 points, where higher scores indicate greater likelihood of RBD (Stiasny-Kolster et al., 2007). The Epworth Somnolence Scale, a simple self-report questionnaire, measures daytime sleepiness levels on a 0–24-point scale, with higher scores indicating more pronounced drowsiness (Johns, 1991). Participants with a score greater than 9 were considered to exhibit signs of daytime sleepiness.

### 2.3 Statistical analysis

To evaluate the longitudinal impact of sleep disturbances on cognitive function, we first applied a linear mixed model to analyze the repeated-measures data. Building upon these analyses, we further employed the lavaan package in R to construct structural equation models (SEM) to test the mediating role of depressive symptoms in the association between sleep disturbances and cognitive outcomes.

Model Directionality Specification:

Given the longitudinal design of the PPMI cohort, which spans from baseline to 60 months, and consistent evidence that excessive daytime sleepiness and REM sleep behavior disorder typically precede significant cognitive decline, we specified a unidirectional path model based on temporal sequence and pathophysiologically grounded hypotheses from prior literature: sleep disturbance → depressive symptoms → cognitive function. This directional assumption aligns with the well-established clinical progression pattern in PD, wherein non-motor symptoms commonly emerge before overt cognitive deterioration, thereby minimizing potential bias from reverse causality (Puig-Davi et al., 2024; Junho et al., 2018; Amara et al., 2017).

Variable definition and handling:

Cognitive function was indexed by the total score of the Montreal Cognitive Assessment. This global metric was selected because early cognitive decline in PD typically manifests as diffuse, multi-domain impairment rather than isolated deficits in specific domains (e.g., memory or executive function alone), enhancing sensitivity for detecting mild cognitive impairment (MCI).

Sleep disturbances were operationalized using two continuous measures representing distinct dimensions:

Daytime sleepiness: assessed via the raw total score of the Epworth Sleepiness Scale (ESS).

RBD symptom severity: measured by the raw total score of the REM Sleep Behavior Disorder Screening Questionnaire (RBDSQ).

Depressive symptoms were evaluated using the raw total score of the 15-item Geriatric Depression Scale (GDS-15).

All core variables, including GDS, ESS, RBDSQ, and MoCA, were entered into the models as continuous, untransformed scores to preserve measurement precision and maximize statistical power.

Mediation model specification:

To comprehensively examine the differential effects of distinct sleep disturbance phenotypes, we constructed two separate SEM mediation models:

Model 1: ESS score (representing EDS) as the predictor.

Model 2: RBDSQ score (representing RBD risk) as the predictor.

In both models, GDS score served as the mediator, and MoCA total score as the outcome. Latent variables were identified by fixing the factor loading of the first indicator to 1 (marker variable method). Model parameters were estimated using maximum likelihood estimation (ML).

To enhance the robustness and statistical reliability of mediation testing, we performed 5,000 bootstrap resamples to compute 95% confidence intervals (CIs) and robust standard errors for both direct and indirect effects. A mediation effect was considered statistically significant if the 95% CI for the indirect effect did not include zero (*p* < 0.05).

Handling of race in covariate adjustment:

Race was included as a covariate in all models. Due to the limited number of non-White participants (including Black, Asian, and Hispanic individuals; *n* = 25), fine-grained racial/ethnic subgroup analyses were not feasible. Accordingly, race was dichotomized as White (reference group, coded as 1) versus non-White (coded as 2). While this simplification acknowledges the heterogeneity within non-White groups in terms of cultural, socioeconomic, and neurocognitive profiles, it represents a necessary statistical compromise given sample size constraint.

Temporal structure:

The time metric “0–4” in the models corresponds to five key longitudinal assessment waves: baseline (=0), 12 months (=1), 24 months (=2), 36 months (=3), and 48 months (=4), capturing the dynamic trajectories of the study variables over time.

Model Visualization:

All SEM results are presented as path diagrams:

Solid lines (——) indicate statistically significant paths (*p* < 0.05).

Dashed lines (− − -) represent non-significant paths (*p* ≥ 0.05).

Path coefficients are standardized *β* values, labeled directly on the respective paths.

Abbreviations used in the figures are defined as follows:

DEP: Depression.

GDS: Geriatric Depression Scale.

RBD: REM Sleep Behavior Disorder.

RBDSQ: REM Sleep Behavior Disorder Screening Questionnaire.

EDS: Excessive Daytime Sleepiness.

ESS: Epworth Sleepiness Scale.

COG: Cognitive Function.

MoCA: Montreal Cognitive Assessment.

## 3 Results

### 3.1 Baseline information of participants

A total of 450 patients with Parkinson’s disease were included, with a median age of 62.1 years (32.2–84.9 years) at enrollment (Table 1).

**Table 1:** Baseline information.

Variable	Quantity
Age, y (±SD)	61.4 ± 9.7
Race, n (%)	
White	425 (94.4%)
Black	3 (0.7%)
Asian	5 (1.1%)
Other	17 (3.8%)
Sex	
Male, n (%)	274 (61%)
Female, n (%)	176 (39%)
Years of education, y (±SD)	16.0 ± 3.2
Moca, y (±SD)	26.4 ± 2.4
GDS-15, y (±SD)	2.8 ± 2.3
Rem, y (±SD)	4.3 ± 2.9
ESS, y (±SD)	6.8 ± 4.3

Moca, Montreal Cognitive Assessment; GDS-15, 15-item Geriatric Depression Scale; REM, Sleep Behavior Disorder Screening Questionnaire (RBDSQ) total score; ESS: Epworth Sleepiness Scale Score.

### 3.2 Linear mixed model analysis

A linear mixed model was constructed to investigate the effects of sleep and depression on cognition, accounting for individual heterogeneity. The random interception and time slope were used to capture individual differences in cognitive scores during each patient’s follow-up. Fixed effect variables included: Gds, Sex, Race, Educ, Rem, and Ess.

After controlling race, gender, and years of education as covariates, the severity of depressive symptoms showed a significant negative correlation with cognitive function (MoCA scores), indicating that each additional point in GDS corresponds to a decrease of approximately 0.115 points in MoCA scores. Daytime sleepiness (ESS) was also significantly associated with cognitive decline, while the impact of rapid eye movement sleep disorder was not statistically significant (see Table 2). Male participants exhibited significantly lower MoCA scores than females (*β* = −0.557, *p* = 0.023). Non-white populations demonstrated lower cognitive scores compared to white populations (*β* = −2.675, *p* < 0.001). Years of education (EDUCYRS) showed a protective effect on cognitive function (*β* = 0.165, *p* < 0.001). The model revealed substantial inter-individual variation in cognitive function but no significant multicollinearity issues (Table 2).

**Table 2:** Associations between sleep, depression, and cognitive function (MoCA) in PD patients: results from linear mixed models.

Variable	Score range	Unstandardized estimate (SE)	Standardized *β* (95% CI)	*p*
GDS	0 ~ 15	−0.115 (0.022)	−0.104 [−0.144, −0.065]	<0.001
Ess	0 ~ 24	−0.046 (0.016)	−0.062 [−0.103, −0.021]	0.003
Rem	0 ~ 13	−0.035 (0.023)	−0.032 [−0.074, 0.010]	0.130
Race (Non-White)		−2.675 (0.502)	−0.832 [−1.139, −0.526]	<0.001
Sex (Male)		−0.557 (0.245)	−0.173 [−0.323, −0.024]	0.023
EDUCYRS	Years	0.165 (0.034)	0.182 [0.108, 0.255]	<0.001

Model adjusted for race, sex, and educational attainment.

### 3.3 Analysis of mediation effect

**Figure 2:**
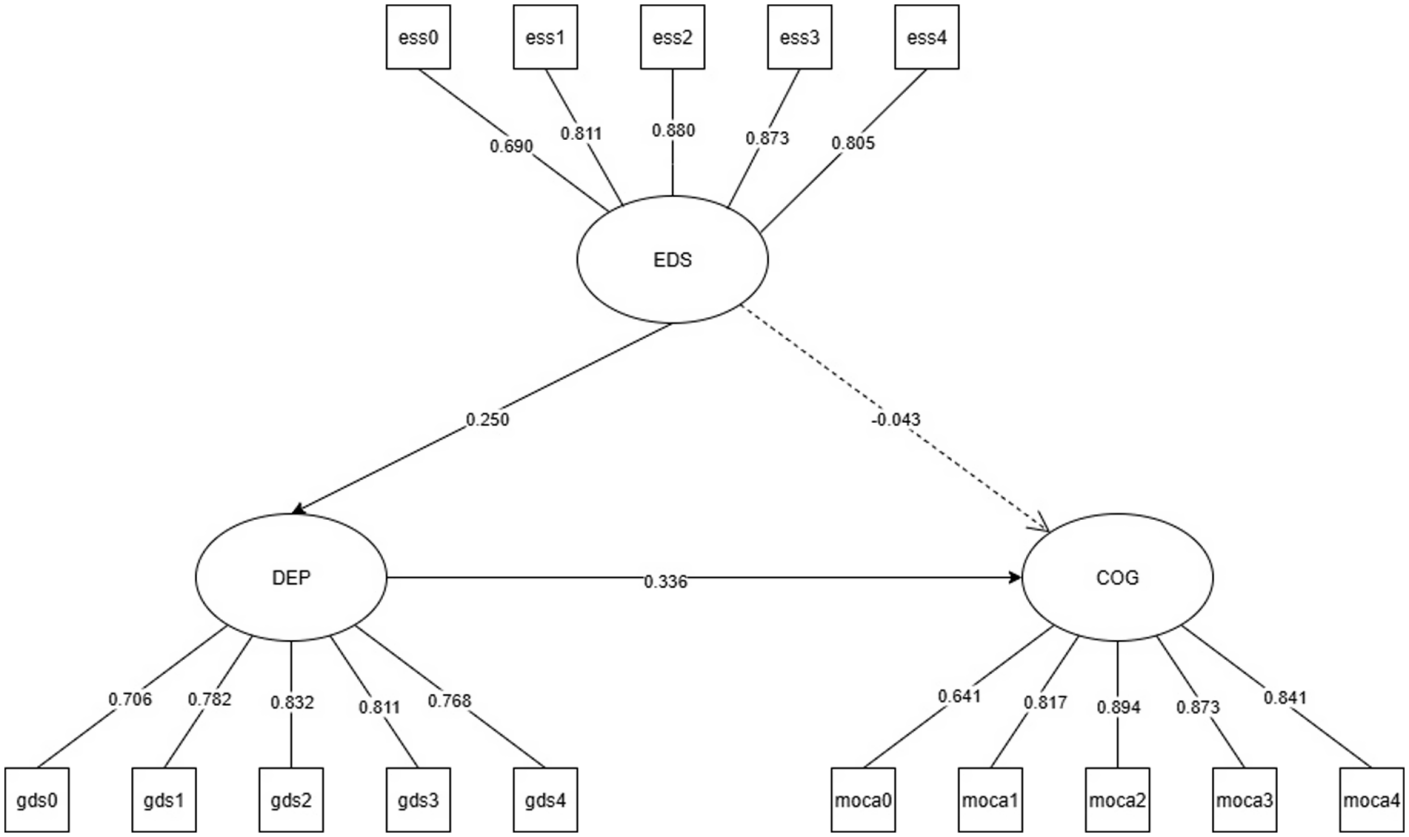
Model 1 A pathway model for daytime excessive sleepiness (EDS) to fully mediate cognitive function (COG) through depressive symptoms (DEP).

**Figure 3:**
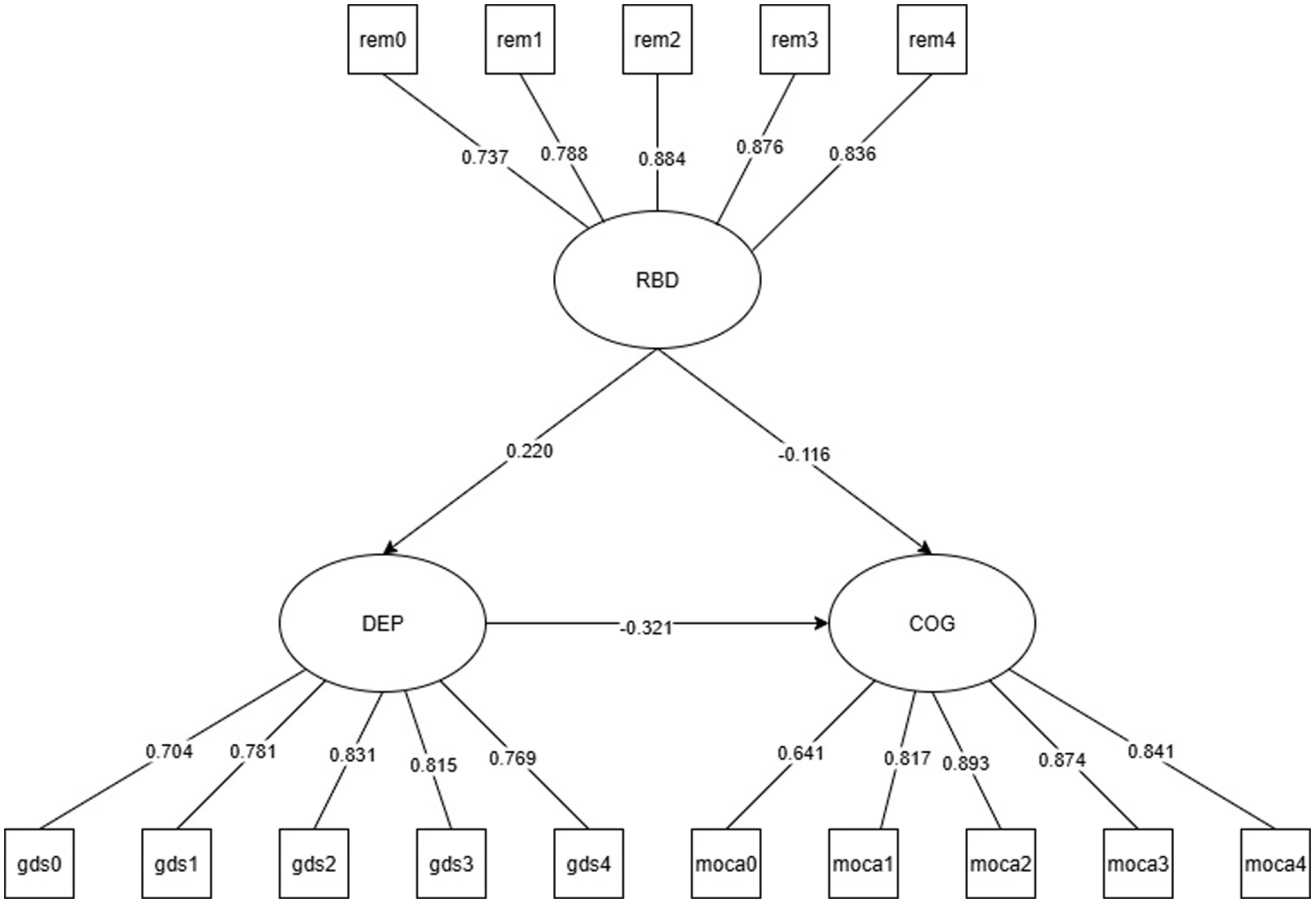
Model 2 REM sleep disorder (RBD) mediated the path model of cognitive function (COG) through depressive symptoms (DEP).

The mediation effect was tested using structural equation modeling with R’s lavaan package, establishing two well-fitted models: Model 1 (CFI = 0.954; TLI = 0.944; RMSEA = 0.071; SRMR = 0.045) and Model 2 (CFI = 0.963; TLI = 0.955; RMSEA = 0.065; SRMR = 0.041). The results are presented in Figure 2 for Model 1 and Figure 3 for Model 2.

The measurement model in Model 1 demonstrated ideal validity for all latent variables ‘observed indicators (standardized factor loadings: 0.641–0.894, *p* < 0.001). Structural modeling analysis revealed that EDS significantly positively predicted DEP (*β* = 0.250, *p* < 0.001), while DEP exerted a significant negative influence on COG (*β* = −0.336, *p* < 0.001). After controlling for DEP, the direct effect of EDS on COG became insignificant (*β* = −0.043, *p* = 0.511). Bootstrap tests further confirmed that EDS’s indirect effect on COG through GDS was statistically significant (*β* = −0.084, 95%CI [−0.097, −0.028]), accounting for 66.1% of the total effect (total effect *β* = −0.127, *p* = 0.062). The results indicate that EDS’s negative impact on COG is entirely mediated by DEP, suggesting that daytime hypersomnia primarily damages cognitive function by exacerbating depressive symptoms (Table 3).

**Table 3:** Mediation effect analysis for Model 1 (EDS → DEP → COG).

Pathway	Standardized coefficient (β)	Standard error (SE)	*p*	95% bootstrap CI	Proportion of total effect
Direct effect					
EDS → COG (controlling for DEP)	−0.043	0.065	0.511		
Indirect effect					
EDS → DEP → COG	−0.084	0.018	<0.001	[−0.097, −0.028]	66.1%
Total effect					
EDS → COG (without controlling DEP)	−0.127	0.068	0.062	[−0.175, 0.001]	100%

The measurement model in Model 2 demonstrated good validity for all latent variables’ observed indicators, with standardized factor loadings ranging from 0.641 to 0.893 (*p* < 0.001), indicating the reliability of the measurement tool. Structural path analysis revealed that RBD abnormalities not only directly negatively affect cognitive function (*β* = −0.116, *p* = 0.035) but also impair cognitive function by significantly increasing depressive levels (*β* = 0.220, p < 0.001). Mediation effect tests showed that REM abnormalities exerted a significant indirect effect on cognition through depression (*β* = −0.071, 95%CI [−0.110, −0.029], *p* = 0.002), accounting for 38.2% of the total effect (total effect *β* = −0.186, *p* = 0.001). The robustness of the results was validated through 5,000 Bootstrap samples. The study findings indicate that RBD’s direct impact on COG is independent of the DEP pathway (Table 4).

**Table 4:** Mediation effect analysis for Model 1 (RBD → DEP → COG).

Pathway	Standardized coefficient (β)	Standard error (SE)	*p*	95% bootstrap CI	Proportion of total effect
Direct effect					
RBD → COG	−0.116	0.055	0.035		
Indirect effect					
RBD → DEP → COG	−0.071	0.021	<0.002	[−0.110, −0.029]	38.2%
Total effect					
RBD → COG	−0.186	0.056	0.001	[−0.225, −0.072]	100%

## 4 Discussion

This longitudinal PD study identifies distinct pathophysiological pathways linking two sleep disturbances, excessive daytime sleepiness and REM sleep behavior disorder, differentially impact cognitive function. These findings illuminate heterogeneity in PD cognitive decline and provide a foundation for precision interventions based on sleep phenotype.

In PD, EDS primarily impairs cognition through depressive symptoms, accounting for 66.1% of its total effect, robustly validating the “sleep–mood–cognition” cascade in PD. This aligns with PD-specific neuroanatomy: EDS stems from early degeneration of arousal-regulating regions (pedunculopontine nucleus (PPN), locus coeruleus (LC), hypothalamic orexin neurons) characteristic of Braak stages 2–3 with documented neuronal loss (Ross et al., 2012; Ogawa et al., 2022; Fronczek et al., 2007; Buddhala et al., 2015).

PD depression reflects specific neurodegeneration: degeneration of the dorsal raphe nucleus serotonergic system and reduced dopaminergic/noradrenergic innervation of prefrontal-limbic circuits (e.g., vmPFC-amygdala), evidenced by neuroimaging (Politis and Niccolini, 2015; Muñoz et al., 2020). Consequently, EDS impairs cognition via: (1) PPN-LC-thalamocortical dysfunction diminishing arousal and attention (Witzig et al., 2025; Geibl et al., 2024); (2) exacerbated DRN-prefrontal dysregulation impairing executive function (Li et al., 2025; Riga et al., 2024). Prospectively, baseline EDS increases depression risk and PPN dysfunction predicts worsening EDS and depression.

Conversely, RBD impacts cognition both through depression and via a significant direct negative effect (*β* = −0.116, *p* = 0.035, persisting after adjustments), reinforcing its role as a high-risk PD cognitive biomarker. This reflects RBD’s origin in early *α*-synuclein pathology within brainstem nuclei (e.g., subcoeruleus nucleus) (García-Lorenzo et al., 2013). Critically, despite minimal early hippocampal Lewy pathology, RBD-related brainstem pathology may propagate anterogradely, disrupting hippocampal-entorhinal connectivity and potentially causing memory deficits before overt atrophy (Fitzgerald et al., 2025).

Our findings are consistent with and extend the existing literature. On one hand, the study provides empirical support within a PD population for the applicability of the depression mediation model (Xu et al., 2025), confirming the central mediating role of depressive symptoms in cognitive impairment associated with excessive daytime sleepiness. On the other hand, we are the first to isolate a direct effect of RBD on cognition in a longitudinal PD cohort, moving beyond prior oversimplified interpretations that attributed RBD’s cognitive impact solely to comorbid depression or general sleep fragmentation.

It is crucial to emphasize that although RBD occurs across multiple synucleinopathies (e.g., multiple system atrophy), its association with cognitive decline exhibits greater predictive specificity within the PD context. This is because the cognitive trajectory in PD is primarily driven by dysfunction in subcortical and limbic circuits, rather than by the temporoparietal cortical atrophy characteristic of Alzheimer’s disease. Therefore, framing our results within a broad “neurodegenerative disease” paradigm would obscure their PD-specific pathophysiological significance; accordingly, we explicitly situate our findings within the PD pathological spectrum.

From a clinical perspective, our results strongly support a subtype-specific, stratified management approach to sleep disturbances in PD:

For patients predominantly affected by EDS, interventions should focus on optimizing nocturnal sleep architecture (e.g., timing adjustments of dopaminergic medications, treatment of comorbid sleep apnea), monitoring metabolic parameters (e.g., glucose and lipid profiles), and concurrently screening for and treating depression.

For those diagnosed with RBD, earlier initiation of cognitive surveillance is warranted, alongside exploration of neuroprotective strategies, such as cautious use of melatonin or clonazepam, or, prospectively, disease-modifying therapies targeting *α*-synuclein pathology.

Furthermore, the protective effect of higher educational attainment, interpreted as a proxy for cognitive reserve, suggests that non-pharmacological interventions (e.g., cognitive training, social engagement) effectively buffer the adverse cognitive impact of sleep disturbances, offering a novel avenue for comprehensive PD care.

In summary, within a PD-specific framework, this study delineates distinct mechanistic pathways through which EDS and RBD contribute to cognitive decline. These insights not only enrich integrative models linking non-motor symptoms to cognitive impairment in PD but also lay the groundwork for future personalized interventions grounded in biopsychosocial risk profiling. Subsequent research should integrate longitudinal multimodal biomarkers with interventional trials to further evaluate the reversibility of these pathways and their potential as therapeutic targets.

Several limitations merit acknowledgment. First, despite the longitudinal design strengthening temporal inference, the observational nature of the study precludes causal conclusions. The directional paths estimated by SEM reflect statistically ordered associations; bidirectional feedback between depression and cognition (Yasugaki et al., 2025; Fang et al., 2019; de Bergeyck and Geoffroy, 2023; Siwecka et al., 2025), or shared latent factors such as neuroinflammation or monoaminergic dysregulation, cannot be ruled out. Second, sleep disturbances were assessed using self-report instruments (e.g., ESS, RBDSQ) without objective confirmation via polysomnography (PSG), potentially leading to underestimation of RBD prevalence or misattribution of EDS to other causes (e.g., medication side effects). Third, the absence of cerebrospinal fluid or neuroimaging biomarkers, such as DAT-SPECT, hippocampal volumetry on MRI, or *α*-synuclein seed amplification assays, limits direct validation of the underlying neuropathological mechanisms.

## 5 Conclusion

Using linear mixed models and structural equation modeling, this study delineates divergent mechanisms linking sleep disturbances to cognitive decline in Parkinson’s disease. Depressive symptoms and EDS were both significantly associated with worse cognitive performance, with depression mediating the majority of EDS’s effect. In contrast, RBD exerted both a direct detrimental impact on cognition and an indirect effect partially mediated by depression. Importantly, these statistical associations do not imply causation. The observed pathways likely reflect interconnected neurodegenerative processes involving brainstem, limbic, and cortical networks affected by *α*-synuclein pathology. Notably, higher educational attainment conferred cognitive protection, supporting the cognitive reserve hypothesis in PD. Collectively, these findings underscore the need for sleep-disorder–specific, biopsychosocially informed strategies to identify individuals at high risk for cognitive decline and to develop targeted interventions within the PD framework.

## Data Availability

The original contributions presented in the study are included in the article/supplementary material, further inquiries can be directed to the corresponding author.

## Footnotes

1
https://www.ppmi-info.org/access-data-specimens/download-data
